# Clinicopathological and Molecular Findings in a Case of Canine *Anaplasma phagocytophilum* Infection in Northern Italy

**DOI:** 10.1155/2014/810587

**Published:** 2014-06-05

**Authors:** Francesco Dondi, Samanta Russo, Chiara Agnoli, Nicola Mengoli, Andrea Balboni, Alberto Alberti, Mara Battilani

**Affiliations:** ^1^Department of Veterinary Medical Sciences, Alma Mater Studiorum, University of Bologna, Via Tolara di Sopra 50, 40064 Ozzano dell'Emilia, Italy; ^2^“Dr Nicola Mengoli” Veterinary Clinic, Via Di Mezzo 51, 40060 Toscanella di Dozza, Italy; ^3^Department of Veterinary Medicine, University of Sassari, Via Vienna 2, 07100 Sassari, Italy

## Abstract

A documented case of canine granulocytic anaplasmosis coupled with the molecular characterization of the etiological agent is reported for the first time in Northern Italy. The patient showed nonspecific clinical signs such as fever and weight loss. The most relevant clinicopathological findings were thrombocytopenia, hypoalbuminemia, and normal azotemic proteinuria consistent with glomerular diseases. Blood smear examination revealed the presence of intracytoplasmatic inclusions in neutrophils associated with high positive serology for *Anaplasma phagocytophilum*. PCR analysis and sequencing of the amplicon confirm serological diagnosis of *A. phagocytophilum*. Phylogenetic analysis evidenced that the detected bacterial strain belongs to the *A. phagocytophilum* Europe 1 lineage. Data indicates that *A. phagocytophilum* circulates in natural environments of Emilia-Romagna region (Northern Italy) and its prevalence in dogs could be underestimated because the clinical signs are frequently nonspecific and a certain diagnosis requires the combination of clinicopathological and molecular assays. Pets living in this area should be regularly monitored and treated for ectoparasites to minimize health risks for humans and pets. Also, surveillance of *A. phagocytophilum* should be improved in Northern Italy and canine anaplasmosis should be considered in differential diagnosis of persistent proteinuria.

## 1. Introduction

Tick-borne rickettsiae in the genera* Ehrlichia* and* Anaplasma* are emerging pathogens with both veterinary and human health implications [1]. Recent taxonomic changes have reclassified the families Rickettsiaceae and Anaplasmataceae in the order Rickettsiales with the unification of some species of* Ehrlichia* under the unique species* Anaplasma phagocytophilum* [2].* A. phagocytophilum* is maintained in nature in an enzootic cycle including* Ixodes* spp. ticks as the main competent vector and a wide range of mammalian species acts as reservoir and source of infection for domestic animals and humans [1]. In Italy,* A. phagocytophilum* has been widely detected in ticks [3, 4], wild ungulates [5], domestic animals including pets [6–8], and humans [9].* A. phagocytophilum* in dogs causes nonspecific clinical and clinicopathological findings as fever, anorexia, weight loss, and thrombocytopenia [10]. Additional diagnostic procedures in clinical practice are frequently necessary in order to reach a correct diagnosis of canine anaplasmosis.

In this study, we report a case of canine granulocytic anaplasmosis documented by complete clinical and clinicopathological description and by molecular investigation of the etiological agent, in Northern Italy. Furthermore, two asymptomatic dogs sharing the same household with the reported case and showing serological evidences of anaplasmosis were evaluated.

## 2. Materials and Methods

### 2.1. Hematology, Chemistry, Urinalysis, Serology, and Vector-Borne Pathogens Screening

Hematology and chemistry were performed at days 0, 3, 10, and 30 of illness using an automated hematology system (ADVIA 2120, Siemens Healthcare Diagnostics, Tarrytown NY, USA) and a chemistry analyzer (AU 400, Olympus/Beckman Coulter, Munich, Germany), respectively. Blood smear Romanowsky staining and microscopic evaluation were performed. C-reactive protein (CRP) (CRP OSR6147, Olympus/Beckman Coulter, Munich, Germany) and urinary protein to creatinine ratio (UPC) and urinary albumin to creatinine ratio (UAC, Microalbumin OSR6167, Olympus/Beckman Coulter, Munich, Germany) were performed as previously reported [11, 12]. Indirect immunofluorescent antibody (IFA) titers for* A. phagocytophilum*,* Ehrlichia canis*, and* Leishmania* spp. were quantified (MegaScreen FLUOANAPLASMA ph, MegaScreen FLUOEHRLICHIA c., MegaCor Diagnostik, Höerbranz, Austria; FLUOLEISH, Virbac, Carros, France). Titers were considered as indicative of infection if >1 : 40. Microscopic agglutination test (MAT) for* Leptospira* spp. was performed at the Animal National Leptospirosis Referral Laboratory (Istituto Zooprofilattico Sperimentale della Lombardia e dell'Emilia-Romagna, Bologna, Italy); antibody titers were determined against 8 serogroups (Australis, Ballum, Canicola, Grippotyphosa, Icterohemorrhagiae, Pomona, Sejroe, and Tarassovi). The dog was also tested for* Dirofilaria immitis* antigen (SNAP Heartworm RT Test, IDEXX laboratories Inc., Westbrook, USA).

### 2.2. Molecular and Phylogenetic Analysis

Genomic DNA extraction from EDTA-blood samples was performed using a commercial kit (NucleoSpin Tissue Mini Kit, Macherey-Nagel, Düren, DE). DNA amplification was implemented with conventional PCR as previously described [13]: a couple of degenerate primers, targeting a fragment of the heat shock protein (groEL), was used to detect DNA from all known* Ehrlichia* and* Anaplasma* spp. A recombinant plasmid containing a portion of the groEL gene of* A. phagocytophilum* was used as positive control [8]. Amplified DNA product was purified and directly sequenced. The nucleotide sequences obtained were assembled and analyzed by BLAST web interface (http://blast.ncbi.nlm.nih.gov/Blast.cgi). A nucleotide-nucleotide search (BLASTN), performed with the default settings, has allowed us to reassemble the sequence obtained in this study to* A. phagocytophilum*. The sequence was submitted to the GenBank database with accession number KF778380 (strain 393/2013). Multiple alignments with reference sequences available from the GenBank nucleotide database were generated using the CLUSTAL W method [14] and phylogenetic analysis was performed using maximum likelihood (ML) methods implemented on MEGA version 5.2.2 [15].

## 3. Results 

### 3.1. Case Report

**Figure 1:**
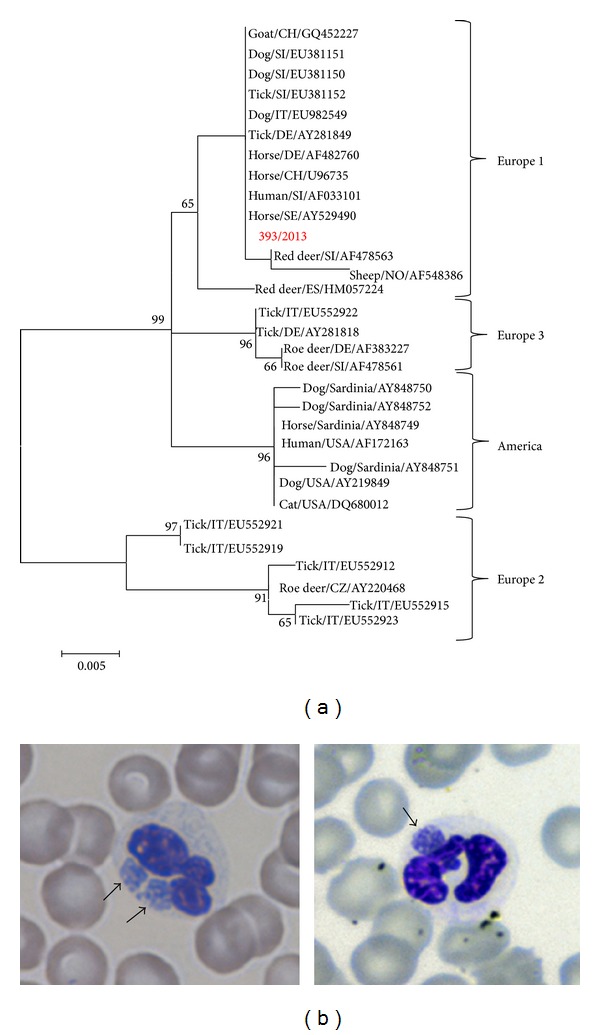
(a) ML tree based on the GroEL alignment. The following reference* A. phagocytophilum* strains detected in several hosts from various parts of the world were obtained from GenBank and included in the molecular analysis:* America lineage*, accession numbers: AY848750; AY848752; AY848749; AF172163; AY848751; AY219849; DQ680012;* Europe 1 lineage*, accession numbers: AF033101; GQ452227; EU381151; EU381150; EU381152; EU982549; AY281849; AF482760; U96735; AY529490; AF478563; AF548386; HM057224;* Europe 2 lineage*, accession numbers: EU552921; EU552919; EU552912; AY220468; EU552915; EU552923; and* Europe 3 lineage*, accession numbers: EU552922; AY281818; AF383227; AF478561. To assess support for individual nodes, bootstrap resampling values were estimated with 1000 replicates. (b) Blood smear (May-Grunwald Giemsa staining) showing* A. phagocytophilum* morulae (black arrows) in the cytoplasm of neutrophil granulocytes of Case 1.

A 12-year-old spayed female English Setter dog (Case 1) was presented to the authors' veterinary teaching hospital (VTH) following a 4-day history of anorexia, weakness, and polyuria/polydipsia. The referring practitioner reported no previous signs of illness or recent treatments and a past occasional exposure to ticks. Routine vaccinations (including Leptospira Canicola and Icterohemorrhagiae serovars), heartworm, and flea/tick chemoprophylaxis were current. The dog was mostly an outdoor pet and was usually used for hunting activities; however, no travels outside the region (Emilia-Romagna, Northern Italy) were reported. Physical examination revealed hyperthermia (39.8°C), tachypnea (40 breathe/min), and tachycardia (80 pulse/min). Thoracic radiographs and abdominal ultrasound were unremarkable. Laboratory variables results are reported in Table 1. At the blood smear examination a mean of 10% of neutrophils presented cytoplasmic inclusions that were characterized by blue-violet aggregates of punctiform bodies, coherent with morulae of* A. phagocytophilum* (Figure 1(b)). Final interpretation of the blood smear was mild to moderate leukopenia, severe thrombocytopenia, and suspected granulocytic anaplasmosis. All serological tests as well as the* Dirofilaria immitis* antigen test resulted in negative except for* A. phagocytophilum* IFA titer (≥1 : 1280).

The dog was hospitalized and immediately treated with oral doxycycline (Vibravet, 10 mg/kg q24, for 28 days). No bleeding tendency was detected and the platelet count returned within the reference interval (WRI) 48 hours after starting the therapy. Clinical signs and clinicopathological abnormalities disappeared completely at day 10, with the exception of proteinuria and albuminuria. At day 30 proteinuria persisted and oral enalapril (Enacard, 0.5 mg/kg q12) was started. No renal biopsy was performed. To date (day 305) the dog is completely asymptomatic; however, it is still presents mild persistent proteinuria and albuminuria.

Further two asymptomatic dogs (cases 2 and 3), both English Setter, female, 7-year-old, sharing the same household of the reported clinical case, were referred to the authors' VTH and the same diagnostic protocol reported above was applied. They showed only high* A. phagocytophilum* IFA titer (≥1 : 1280) and were treated with oral doxycycline (Vibravet, 10 mg/Kg q24, for 28 days). Other clinicopathological variables were WRI.

### 3.2. Molecular and Phylogenetic Analysis

Positive PCR product of the expected size of 600 bp, corresponding to a fragment of the heat shock protein (groEL) gene was observed for Case 1. Cases 2 and 3 resulted in negative. A nucleotide sequence of 467 bp was obtained from the amplicon detected in Case 1. The partial groEL gene sequence obtained was analyzed by BLAST web interface and it resulted in having 100% of identity with analogous sequences of* A. phagocytophilum* present in GenBank.

The nucleotide alignment showed complete identity between strain 393/2013 and several* A. phagocytophilum *strains detected in various hosts and countries (Horse/SE/AY529490, Human/SI/AF033101, Horse/CH/U96735, Horse/DE/AF482760, Tick/DE/AY281849, Dog/IT/EU982549, Tick/SI/EU381152, Dog/SI/EU381150, Dog/SI/EU381151, and Goat/CH/GQ452227). Identity values among the 393/2013 strain and the reference strains ranged from 92 (Dog/Sardinia/AY848751) to 99,7% (Red_deer/SI/AF478563). Phylogenetic tree showed four main clusters, supported by significant bootstrap values, consistent with previous observation and with the accepted nomenclature (Figure 1(a)) [5, 8]. The 393/2013 strain is included in the cluster Europe 1, containing* A. phagocytophilum* strains detected in various hosts, human included. The mean distance calculated with MEGA software between Europe 1 versus other groups ranged from 0.063 (Europe 1 and Europe 2) to 0.017 (Europe 1 and America).

## 4. Discussion

In Italy,* A. phagocytophilum* infection was detected in humans and wild and domestic animals [5–7, 9]; a few reports have documented the evidence of* A. phagocytophilum* infection in nonruminant domestic animals such as cats, dogs, and horses [8, 16]. Most cases of canine granulocytic anaplasmosis were reported in Europe [17–19]; however, in Italy just one case was clinically described (Sicily, Southern Italy) and documented by molecular characterization of the bacterial strain [20]. Natural infection by* Anaplasma* spp. in pet animals frequently goes undetected, because the disease may be subclinical or clinical findings are nonspecific: often, the only signs are fever, depression, and weight loss, and the most common laboratory findings is thrombocytopenia [10]. In clinical practice, diagnosis of anaplasmosis in dogs should be accomplished by combining history, clinical signs, and clinicopathological analysis, including identification of morulae-containing granulocytes on blood smear, serology, and PCR. Diagnostic assays to detect* Anaplasma* spp. infection, however, present some limitations mainly due to short duration of bacteremia and chronic phase of infection.

In this report, we documented clinical and clinicopathological manifestations of* A. phagocytophilum *infection as well as molecular characterization of the bacterial strain detected in a dog. The clinical signs reported in Case 1 supported the diagnosis of infectious tick-borne disease; however, they were nonspecific. Clinicopathological findings in combination with granulocytic morulae, high IFA titer against* A. phagocytophilum*, and even PCR results, allowed clinicians to confirm the etiology. Interestingly, clinical presentation of Case 1 was compatible with a subclinical nonazotemic proteinuric renal disease probably sustained by an infection-associated glomerulopathy as previously suggested [21]. Molecular characterization of the* A. phagocytophilum* strain 393/2013 showed that it belongs to Europe 1 lineage, according to the nomenclature introduced by other authors [8]. Phylogenetic tree showed a clear separation of the strains in European and American lineages, as described previously [5, 8], with a strong statistical support for a partitioning of strains based on sampling location. The nucleotide sequence of 393/2013, in the fragment of groEL gene sequenced, is identical to* A. phagocytophilum *strains detected in various hosts (humans, horses and ticks) and geographical areas, as well as it is identical to the sequence EU982549 available on GenBank, an* A. phagocytophilum *isolate detected in the pleural fluid of dog in Emilia-Romagna region (Northern Italy). The epidemiological significance of genetic variants of* A. phagocytophilum *is poorly understood, but previous studies showed that the Europe 2 genotype was associated with roe deer, whereas the Europe 1 genotype was associated with a wider host range, including both domestic and wild animals [5]. The genetic characteristics do not seem to be clinically or ecologically meaningful and multiple unique strains of* A. phagocytophilum *with distinct host tropisms can circulate in the same geographic area [22]. In order to understand the risk factors associated with transmission of a tick-borne pathogen to humans and domestic animals, several studies of molecular epidemiology were carried out also in Northern Italy. Previous molecular surveys in the Emilia Romagna region have been performed through collecting ticks after having been dragged and removed from wild and domestic animals, dogs included. These surveys have shown the presence of* A. phagocytophilum* [4, 23]. This data, in addition to our case report, demonstrated that* A. phagocytophilum* circulates in the natural environment; therefore, pets living in this area should be regularly monitored and treated for ectoparasites to minimize health risks for humans and pets as well the surveillance of* A. phagocytophilum* should be increased in Northern Italy.

## 5. Conclusion

At the knowledge of the authors, this is the first document case of canine granulocytic anaplasmosis reported in Northern Italy. Our data indicates that* A. phagocytophilum* prevalence in dogs could be underestimated because the clinical signs are frequently nonspecific and a certain diagnosis requires the combination of clinicopathological and molecular assays. Surveillance for* A. phagocytophilum* could be increased also in Northern Italy and canine anaplasmosis should be considered in differential diagnosis of persistent proteinuria.

## Figures and Tables

**Table 1:** Pertinent clinicopathological findings of Case 1.

Variable	Units	T0	T3	T10	T30	Reference interval
Hematology						
RBC	×10^12^/L	5.8	6.4	6.21	6.46	5.5–8.5
Hemoglobin	g/dL	13.3	14.4	14.8	16.0	12.0–18.0
HCT	%	40.5	43.2	44.3	46.5	37.0–55.0
WBC	×10^9^/L	5.4	4.7	10.8	10.1	6.0–17.0
Neutrophils	×10^9^/L	3.7	0.9	7.5	6.5	3.0–12.0
Lymphocytes	×10^9^/L	1.0	2.7	1.8	2.2	1.0–4.8
Monocytes	×10^9^/L	0.3	0.8	0.6	0.6	0.1–1.4
Platelets	×10^9^/L	13	190	537	44.7	16.0–50.0
MPV	fL	21.2	16.3	10.8	9.5	6.6–10.9
PCDW	g/dL	5.7	5.1	7.3	6.2	4.15–8.25
MPC	g/dL	22.7	21.7	20.8	22	17.2–24.4
MPM	pg	3.45	2.85	1.92	1.89	1.83–2.79
PMDW	pg	1.34	1.29	0.77	0.75	0.6–1.1
Chemistry						
Creatinine	mg/dL	0.8		0.53	0.56	0.6–1.3
Urea	mg/dL	29.7		20.37	19.16	18–55
Total protein	g/dL	5.9		5.68	5.69	5.6–7.9
Albumin	g/dL	2.3		2.65	2.91	2.8–3.7
A : G		0.65		0.87	1.05	0.6–1.3
SAP	U/L	291		142	151	42–180
Iron	mcg/dL	65		201	185	50–230
TIBC	mcg/dL	231		299	326	240–440
Saturation	%	28		67	57	30–68
CRP	mg/dL	6.25		0.01		0–0.5
Serum protein electrophoresis						
Albumin	g/dL	2.2				2.63–4.53
*α*_1_-Globulins	g/dL	0.3				0.19–0.34
*α*_2_-Globulins	g/dL	1.3				0.90–1.61
*β*_1_-Globulins	g/dL	0.8				0.27–1.02
*β*_2_-Globulins	g/dL	0.6				0.34–0.87
*γ*-Globulins	g/dL	0.7				0.30–0.78
Urinalysis						
USG		1.040		1.023	1.016	>1.030*
UPC		1.2		1.0	1.2	<0.5
UAC		0.9		0.8	1.4	<0.025

RBC: red blood cells; HCT: hematocrit value; WBC: white blood cells; MPV: mean platelet volume; MPC: mean platelet component; PCDW: platelet concentration distribution width; MPM: mean platelet mass; PMDW: platelet mass distribution width; A : G: albumin to globulin ratio; SAP: serum alkaline phosphatase; TIBC: total iron binding capacity; CRP: C-reactive protein; USG: urine specific gravity; UPC: urine protein to creatinine ratio; UAC: urine albumin to creatinine ratio.

*Canine adequate USG.
